# Recombinant AMA1 Virus-like Particle Antigen for Serodiagnosis of *Toxoplasma gondii* Infection

**DOI:** 10.3390/biomedicines10112812

**Published:** 2022-11-04

**Authors:** Min-Ju Kim, Ki-Back Chu, Jie Mao, Hae-Ji Kang, Gi-Deok Eom, Keon-Woong Yoon, Su-Hwa Lee, Eun-Kyung Moon, Young-Ha Lee, Fu-Shi Quan

**Affiliations:** 1 Department of Biomedical Science, Graduate School, Kyung Hee University, Seoul 02447, Korea; 2 Medical Research Center for Bioreaction to Reactive Oxygen Species and Biomedical Science Institute, Core Research Institute (CRI), Kyung Hee University, Seoul 02447, Korea; 3 Center for Translational Antiviral Research, Georgia State University Institute for Biomedical Sciences, Atlanta, GA 30303, USA; 4 Department of Medical Zoology, School of Medicine, Kyung Hee University, Seoul 02447, Korea; 5 Department of Infection Biology, College of Medicine, Chungnam National University, Daejeon 34134, Korea; 6 Department of Medical Science, College of Medicine, Chungnam National University, Daejeon 34134, Korea

**Keywords:** *Toxoplasma gondii*, AMA1, virus-like particle, serodiagnosis

## Abstract

Toxoplasmosis diagnosis predominantly relies on serology testing via enzyme-linked immunosorbent assay (ELISA), but these results are highly variable. Consequently, various antigens are being evaluated to improve the sensitivity and specificity of toxoplasmosis serological diagnosis. Here, we generated *Toxoplasma gondii* virus-like particles displaying AMA1 of *T. gondii* and evaluated their diagnostic potential. We found that AMA1 VLPs were highly sensitive and reacted with the sera acquired from mice infected with either *T. gondii* ME49 or RH strains. The overall IgG and IgM antibody responses elicited by AMA1 VLPs were substantially higher than those induced by the conventionally used *T. gondii* lysate antigen (TLA). Importantly, AMA1 VLPs were capable of detecting parasitic infection with *T. gondii* RH and ME49 as early as 1 week post-infection, even when mice were exposed to low infectious doses (5 × 10^3^ and 10 cysts, respectively). AMA1 VLPs also did not cross-react with the immune sera acquired from *Plasmodium berghei*-infected mice. Compared to TLA, stronger antibody responses were induced by AMA1 VLPs when tested using *T. gondii*-infected human sera. The sensitivities and specificities of the two antigens were substantially different, with AMA1 VLPs demonstrating over 90% sensitivity and specificity, whereas these values were in the 70% range for the TLA. These results indicated that AMA1 VLPs can detect infections of both *T. gondii* ME49 and RH at an early stage of infection caused by very low infection doses in mice, and these could be used for serological diagnosis of human toxoplasmosis.

## 1. Introduction

*Toxoplasma gondii* is an intracellular parasite and the etiologic agent causing the zoonotic disease toxoplasmosis, which can be transmitted to a wide array of warm-blooded vertebrates by ingestion of cyst-contaminated water, raw meat, or other food products [1]. Currently, toxoplasmosis is one of the most prevalent diseases affecting humans with more than a third of the world’s population estimated to be infected [2]. *T. gondii* infection in humans tends to result in non-specific symptoms in the immunocompetent population and the disease manifestation was also reported to be associated with the parasite’s clonal lineage [3]. However, their presence can lead to devastating consequences in children, pregnant women, the elderly, or immunocompromised individuals. Specifically, in pregnant women, *T. gondii* infection can lead to transmission of the parasite to the fetus which ultimately results in miscarriage or stillbirth. Other symptoms associated with congenital toxoplasmosis include cerebral calcification, retinal choroiditis, hydrocephalus, and others [4]. Due to the severity of its consequences, several countries in Europe screen pregnant women for toxoplasmosis [5]. For these reasons, rapidly and accurately diagnosing toxoplasmosis is critical.

To date, a wide array of toxoplasmosis diagnostic methods have been developed. Imaging techniques such as magnetic resonance imaging or ultrasonography are not specific for *T. gondii* infection, but they are frequently used as initial screening steps to identify the location of the affected area and the extent of the damage done [6]. Molecular approaches to *T. gondii* diagnosis are increasing in popularity. For example, real-time PCR is a highly sensitive method that can detect extremely low concentrations of parasitic nucleic acid. One major advantage of molecular testing methods over conventional serology-based assays is that this method can confirm the presence of parasites in fetuses [7].

Serology-based assays are the most commonly used toxoplasmosis diagnostic method which relies on measuring the serum IgM and IgG antibody responses. Notable examples of these methods include the Sabin and Feldman dye test (DT), enzyme-linked immunosorbent assays (ELISA), chemiluminescence assays (CIA), indirect fluorescent antibody test (IFAT), immunosorbent agglutination assays (ISAGA), the latex agglutination test (LAT), the serum IgG avidity test, and western blotting (WB) [7,8]. In particular, ELISA and CIA are universal methods for measuring *T. gondii*-specific IgG and IgM were used as the basis for commercially manufactured diagnostic kits. However, there are issues with the serological testing methods. Due to the fact that IgM levels persist for several months or even years after infection, this can result in the false-positive classification of chronic infections as acute infections [9]. Parasite-specific IgG can also be assessed using *T. gondii* tachyzoite lysate antigen (TLA), but detection is usually possible after 2 to 3 months [10]. In serological diagnosis, sensitivity and specificity depend on the antigen type. Therefore, various recombinant antigens are currently being assessed to replace TLA-based ELISA and these antigens include surface antigens (SAG), dense granule antigens (GRA), and rhoptry antigens (ROP) [11,12,13].

Although limited in number, several studies have explored the use of virus-like particles (VLPs) as a diagnostic tool. VLP-based ELISA was able to differentiate between West Nile virus and St. Louis encephalitis virus infections [14]. The diagnostic potential of these non-infectious VLPs was also reported in studies involving flavivirus and the Japanese encephalitis virus [15,16]. Yet, VLP-based diagnosis for toxoplasmosis remains unreported to date. Recently, in one study, the diagnostic potential of the recombinant *T. gondii* apical membrane antigen 1 (AMA1) expressed in *Escherichia coli* has been explored [17]. However, prokaryotic expression systems lack post-translation modifications (PTMs) and are subjected to improper folding, inclusion body formation, and codon bias, all of which can imply that the recombinant protein may not be a true representation of the parasitic protein’s native form [18]. Baculovirus-based expression systems use insect cells and proteins expressed this way undergo PTMs that are similar to those of mammalian cells [19] thus more closely reflecting the parasitic protein in their native conformation. This approach would enable more accurate detection of *T. gondii* than prokaryote-derived proteins. Furthermore, baculovirus expression systems can be easily scaled to industrial levels at low costs, as well as being inherently safe for humans [20]. To further assess the serodiagnostic potential of the AMA1, we generated baculovirus-based VLP constructs expressing the *T. gondii* AMA1 in insect cells and evaluated the feasibility of this approach. Specifically, we assessed the murine and human serum response to the *T. gondii* AMA1 VLPs and compared the findings to conventional TLA-based ELISA.

## 2. Materials and Methods

### 2.1. Ethics Statement

All animal experiments in this study were carried out under the guidelines set out by Kyung Hee University IACUC. All experimental protocol involving animals was reviewed and approved by the IACUC (permit number: KHSASP-20-165). Blood collection was performed under mild anesthesia, which was induced and maintained with ketamine hydrochloride and xylazine. All efforts were made to minimize the number of animals used in the experiment as well as their suffering. Human subject research approval was obtained from the Kyung Hee University Institutional Review Board (KHSIRB-20-178(EA)). The need for consent was waived by the ethics committee.

### 2.2. Animals, Parasites, Cells, and Antibodies

*Toxoplasma gondii* RH, ME49, and *P. berghei* ANKA strains were maintained in seven-week-old female BALB/c mice obtained from NARA Biotech (Seoul, Korea) following the methods described previously [21,22]. *Spodoptera frugiperda* Sf9 insect cells were cultured in serum-free SF900-II medium (Invitrogen, Carlsbad, CA, USA) and subsequently used to generate recombinant baculovirus (rBV) and virus-like particles (VLPs). Horseradish peroxidase (HRP)-conjugated goat anti-mouse IgG, IgA, IgM, and goat anti-human IgG antibodies were purchased from Southern Biotech (Birmingham, AL, USA) and Invitrogen (Carlsbad, CA, USA). Influenza-infected sera were acquired by intranasally infecting mice with 50 plaque-forming units (pfu) of the H3N2 influenza virus (A/Hong Kong/1968). Sera were collected 4 weeks post-infection. Monoclonal influenza M1 antibody was purchased from Abcam (Cat# 22396, Cambridge, UK). Toxoplasmosis patient sera (n = 17) were confirmed and provided by Chungnam National University (Daejeon, Korea) and Seoul National University (Seoul, Korea).

### 2.3. Generation of AMA1 VLPs

Cloning of *T. gondii* apical membrane antigen 1 (AMA1) was conducted as previously described [23]. Briefly, AMA1 gene (GenBank: AF010264.1) with *EcoR*I and *Xho*I restriction enzyme sites were PCR-amplified and transformed into pFastBac plasmid. After subsequent cloning into DH10Bac competent cell, bacmid DNA was acquired for rBV and VLP production. The baculoviruses and VLPs expressing AMA1 and influenza M1 were prepared as previously described [23]. In brief, bacmid DNA expressing AMA1 or influenza M1 were transfected into Sf9 cells for AMA1 and M1 rBV construction, respectively. The rBVs in the supernatants were carefully acquired and simultaneously inoculated into a fresh batch of Sf9 cells. Thus, VLPs containing both AMA1 and M1 were generated. Harvested VLPs were ultracentrifuged and purified using the sucrose density gradient. Transmission electron microscopy (TEM) was used to characterize the VLPs.

### 2.4. Infection of T. gondii (ME49, RH) and Plasmodium berghei ANKA Strain

Female BALB/c mice were orally infected with *T. gondii* ME49 (10, 50, 100, 150, 300 cysts per mouse; n = 6 for each infection dose), RH (5 × 10^3^, 1 × 10^4^, or 5 × 10^4^ per mouse), or *P. berghei* (5.0 × 10^3^ per 100 μL in PBS). The ME49 and RH strains of *T. gondii* were inoculated using oral gavage, while *P. berghei* were inoculated through intraperitoneal (IP) injection. Blood samples were collected through retro-orbital plexus puncture. From RH-infected mice, sera were collected at 1 and 2 weeks post-infection (wpi). For ME49 infection, sera were acquired at 1, 2, 4, and 8 wpi. Mice were infected twice with *P. berghei* at 2-week intervals, and blood samples were drawn 1 week after the second infection. Sera were stored at −20 °C until use.

### 2.5. Antibody Response Detection in Sera

Serum antibody responses were evaluated using enzyme-linked immunosorbent assay (ELISA). Briefly, 96-well immunoplates were coated with either AMA1 VLPs or *T. gondii* RH lysate antigen (TLA) at concentrations of 4 μg/mL in carbonate coating buffer. After overnight coating at 4 °C, plates were blocked for 1 h at 37 °C using 0.2% gelatin in PBS with 0.05% Tween-20. A total of twenty human sera samples were acquired, with three from naïve individuals and seventeen from toxoplasmosis-positive patients. Human (n = 20) or mice sera (n = 32) were diluted (1:100 dilution in PBS) and inoculated into respective wells. M1 monoclonal antibodies were diluted 1:2000 in PBS. After incubating the plates for 2 h at 37 °C, 100 HRP-conjugated anti-mouse IgG, IgM, and IgA or anti-human IgG secondary antibodies were inoculated into respective wells (1:2000 dilution in PBS). Following 1 h incubation at 37 °C, o-phenylenediamine (Sigma Aldrich, St. Louis, MO, USA) was dissolved in a citrate substrate buffer with H_2_O_2_. Reactions were stopped with H_2_SO_4_ and optical density readings at 492 nm were measured using a microplate reader (EZ Read 400, Biochrom Ltd., Cambridge, UK). ELISA cut-off value for the serum IgG, IgM, and IgA antibody responses was determined to be a mean OD + 3 standard deviation of negative sera. TLA used in the present study was validated by comparing its mouse serum ELISA results to that of commercialized TLA (MyBioSource, Cat #: MBS568631, San Diego, CA, USA). For human sera, the relative sensitivity and specificity of both TLA and AMA1 VLPs were determined using the formula previously described elsewhere [24]. Briefly, the percentages of relative antigen sensitivity and specificity were calculated as follows: relative sensitivity = true positive case/(true positive + false negative) × 100%; relative specificity = true negative case/(true negative + false positive) × 100%. Receiver operating characteristics (ROC) analysis has been used for the comparison of sensitivity and specificity between TgAMA1 VLPs and TLA.

### 2.6. Statistical Analysis

All parameters were recorded for individuals within all groups. All data were presented as mean ± SD and statistical significances between groups were analyzed by one-way analysis of variance (ANOVA) with Bonferroni’s multiple comparison *post hoc* test or Student’s *t*-test using GraphPad Prism version 6.0 (San Diego, CA, USA). *p*-values (* *p* < 0.05) were considered statistically significant.

## 3. Results

### 3.1. Generation of AMA1 VLPs and TLA

**Figure 1:**
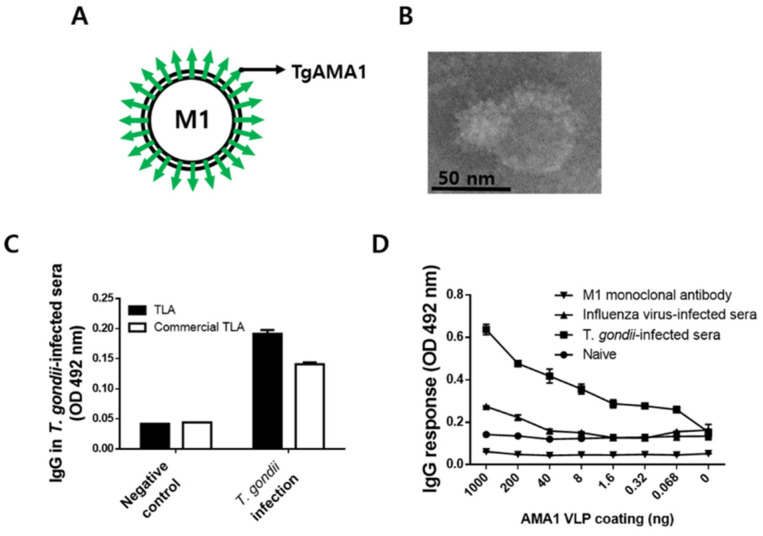
Production of AMA1 VLPs and TLA. Schematic illustration (**A**) and TEM (**B**) for *T. gondii* AMA1 VLPs. AMA1 antigens were expressed on the surface of VLPs, in which influenza M1 was used as a core protein. The TLA we generated was compared with commercial TLA by ELISA using *T. gondii*-infected mouse sera (**C**). The *T. gondii*-specificity of the AMA1 VLPs were evaluated by comparing their antibody response induction against influenza infection-positive sera, naïve sera, and M1 monoclonal antibodies (**D**). Datasets were presented as mean ± SD.

AMA1 VLPs containing influenza M1 and AMA1 of *T. gondii* were generated. The structural components of the VLPs were depicted using an illustration, with AMA1 antigen spikes protruding through the lipid bilayer (Figure 1A). Spherical morphology with minor defects was observed when the VLPs were visualized under TEM (Figure 1B). To confirm that the TLA used in the present study was comparable to that of commercialized TLA, ELISA was performed using naïve and *T. gondii* (ME49)-infected mouse sera (Figure 1C). Antibody responses elicited from naïve and *T. gondii*-infected mice sera were compared using the two antigens. As seen in Figure 1C, the overall antibody responses acquired from both antigens were comparable, thereby validating that the TLA we used in the present study is suitable for use. Furthermore, to evaluate the specificity of the AMA1 VLPs, antibody responses elicited from *T. gondii*-infected sera were compared to those of other control groups (Figure 1D). As expected, neither influenza infection sera nor monoclonal M1 antibody reacted with AMA1 VLPs, thereby confirming their species specificity.

### 3.2. Sera of T. gondii ME49-Infected Mice React Stronger to AMA1 VLPs Than TLA

**Figure 2:**
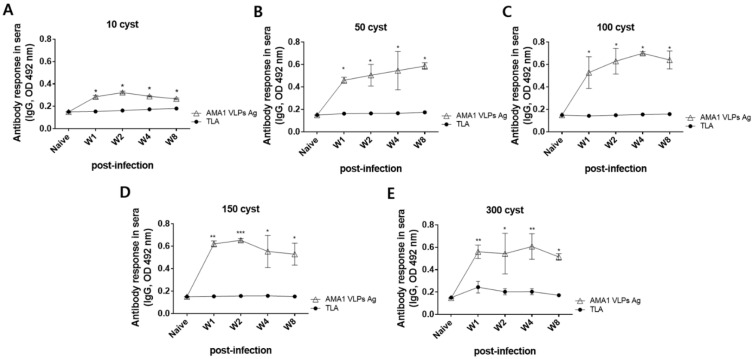
Reactivity of *T. gondii* ME49 infection-induced IgG antibody response to TLA and AMA1 VLPs. Mice were orally infected with 10 (**A**), 50 (**B**), 100 (**C**), 150 (**D**), or 300 (**E**) cysts of the *T. gondii* ME49 strain. Sera were collected at regular intervals to evaluate ME49-specific IgG antibody responses by ELISA against either TLA or AMA1 VLPs. Data are presented as mean ± SD and are representative of the 3 individual experiments performed in triplicates (* *p* < 0.05, ** *p* < 0.01, *** *p* < 0.001 vs. TLA).

Sera of ME49-infected mice were collected at 1, 2, 4, and 8 wpi to assess the antibody responses against AMA1 VLPs and TLA. Differences in antibody responses were noticeable starting at week 1 for all infection doses (Figure 2A–E). IgG responses to AMA1 VLPs were significantly greater than those of TLA at all time points. These durable antibody responses were detected from 1 wpi. Increasing the infection dose resulted in stronger antibody response to AMA1 VLPs, but not to TLA. Evidently, the OD values elicited by sera of 50 or more *T. gondii* ME49 cyst-infected mice were substantially higher than that elicited by sera of 10 cyst-infected mice. While this effect appeared to be dose-dependent at first, further increasing the infection dose to 300 did not result in a profound antibody response.

### 3.3. Robust Antibody Response Is Elicited by T. gondii RH Infection Sera against AMA1 VLPs, but Not TLA

**Figure 3:**
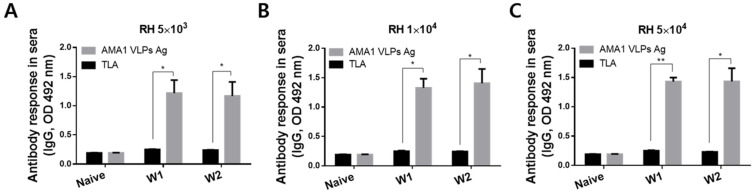
Reactivity of *T. gondii* RH infection-induced IgG antibody to AMA1 VLPs antigen. Mice were orally infected with 5 × 10^3^ (**A**), 1 × 10^4^ (**B**), or 5 × 10^4^ (**C**) tachyzoites of the *T. gondii* RH strain. Sera were collected at 1 and 2 wpi to assess antigen-specific antibody responses. Data are presented as mean ± SD and are representative of the three individual experiments performed in triplicate (* *p* < 0.05, ** *p* < 0.01 vs. TLA).

To confirm that AMA1 VLPs could be used against other clonal lineages, *T. gondii* RH strains were used to infect mice and their sera were collected. Dose-dependent antibody inductions were not detected in RH-infected mice, as antibody responses were similar regardless of infection dose. The sera of RH-infected mice interacted strongly with AMA1 VLPs and this was observed as early as 1 wpi (Figure 3A–C). Specifically, sera interacted significantly greater with AMA1 VLPs than against TLA at both 1 and 2 wpi (* *p* < 0.05, ** *p* < 0.01), irrespective of the infection dose. The overall inductions were strikingly higher than the ME49 positive control sera.

### 3.4. T. gondii ME49-Infected Sera Elicited Durable IgM Antibody Responses against AMA1 VLPs

**Figure 4:**
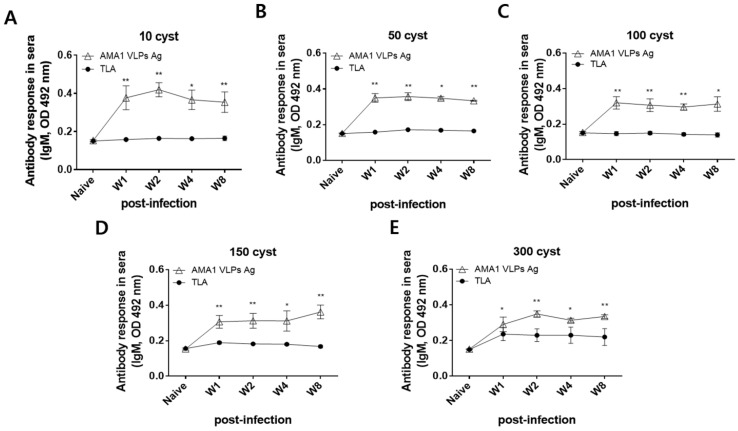
Reactivity of *T. gondii* ME49 infection-induced IgM antibody to AMA1 VLPs antigen. A total of 10 (**A**), 50 (**B**), 100 (**C**), 150 (**D**), or 300 (**E**) *T. gondii* ME49 cysts were inoculated into mice and infection sera were collected at 1, 2, 4, and 8 wpi. IgM responses to both TLA and AMA1 VLPs were assessed via ELISA. Data are presented as mean ± SD and are representative of the three individual experiments performed in triplicate (* *p* < 0.05, ** *p* < 0.01 vs. TLA).

*T. gondii*-specific IgM responses against TLA and AMA1 VLPs were determined using the sera acquired from ME49-infected mice. Antigen-specific IgM levels against TLA were consistent throughout the 8-week infection period. Contrary to this, IgM responses against AMA1 were significantly greater than those elicited against TLA at all time points irrespective of infection dosage (* *p* < 0.05, ** *p* < 0.01). Overall, the IgM antibody responses to AMA1 VLPs were consistently maintained at higher levels throughout the 8-week infection period than those elicited against the TLA (Figure 4A–E).

### 3.5. T. gondii RH Infection Induces Potent IgM Antibody Responses to AMA1 VLPs

**Figure 5:**
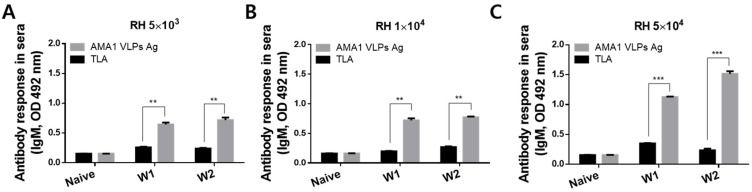
Reactivity of *T. gondii* RH infection-induced IgM antibody to AMA1 VLPs antigen. After orally administering 5 × 10^3^ (**A**), 1 × 10^4^ (**B**), or 5 × 10^4^ (**C**) doses of *T. gondii* RH tachyzoites, sera were collected at 1 and 2 wpi to evaluate *T. gondii*-specific IgM antibody responses. ELISA was performed using coated TLA or AMA1 VLPs as antigens. Data are presented as mean ± SD and are representative of the 3 individual experiments performed in triplicate (** *p* < 0.01, *** *p* < 0.001 vs. TLA).

To investigate the levels of IgM antibody response, the sera from *T. gondii* RH-infected mice were collected at 1 and 2 wpi. Similar to the IgG results, IgM responses from the sera of RH-infected were maintained at significantly high levels until 2 wpi. Compared to IgG responses, IgM levels appeared to be dose-dependent. While differences between 5 × 10^3^ and 1 × 10^4^ were negligible, a marked increase in antibody responses was observed from the sera of mice infected with 5 × 10^4^ *T. gondii* RH (Figure 5A–C). Antibody response to TLA was maintained at similar levels across the 2 weeks of infection.

### 3.6. Specificity of Mouse IgG and IgA Antibody Responses to T. gondii AMA1 VLPs Antigen

**Figure 6:**
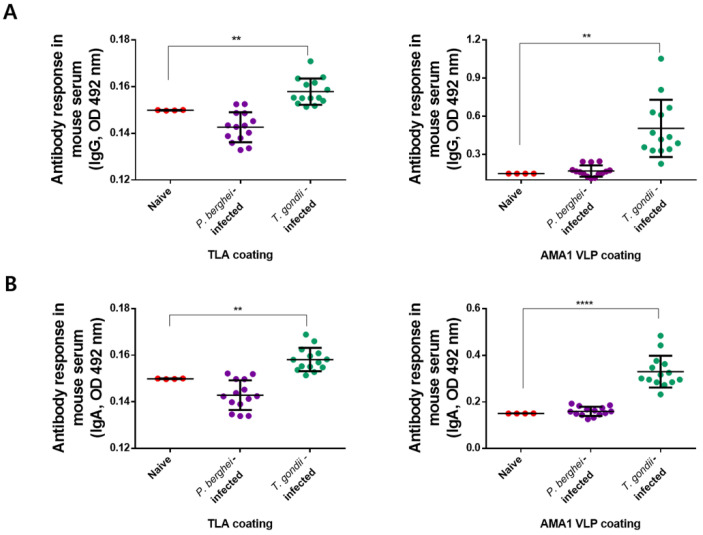
Parasite-specificity of mouse IgG and IgM antibody responses against AMA1 VLPs antigen. To confirm whether the elicited antibody responses against TLA and AMA1 VLPs are highly specific for *T. gondii* but not other Apicomplexan parasites, mice infected with the rodent malaria pathogen *P. berghei* and sera were collected. IgG (**A**) and IgA (**B**) antibody responses against both TLA and AMA1 VLPs were compared using the *P. berghei* or *T. gondii* immune sera via ELISA. Data are presented as mean ± SD and are representative of the three individual experiments performed in triplicate (** *p* < 0.01, **** *p* < 0.0001 vs. Naive).

In order to assess the specificity of the AMA1 VLPs to *T. gondii*, mice were infected with other parasites belonging to the phylum *Apicomplexa* such as *P. berghei* which causes rodent malaria. Sera were collected from *P. berghei*-infected mice and their responses were compared to those of *T. gondii*-infected mice against both TLA and AMA1 VLPs. Cross-reaction did not occur between *P. berghei* and *T. gondii*, as demonstrated by the naïve-like antibody response from *P. berghei*-infected mice sera. On the contrary, significantly increased IgG response against TLA was observed from the sera of *T. gondii*-infected mice (Figure 6A). Identical findings were observed when the sera were reacted using AMA1 VLPs. However, the differences between the naïve and *T. gondii*-infected sera were much more distinct and noticeable. These findings were also observed from IgA antibody responses (Figure 6B). As with IgG, *P. berghei* infection-positive sera did not interact with TLA or AMA1 VLPs. Sera of *T. gondii*-infected mice interacted with TLA and elicited significantly higher parasite-specific IgA response. This was also observed when evaluated using the *T. gondii* AMA1 VLP as a coating antigen.

### 3.7. T. gondii-Infected Human Sera Induce Stronger Antibody Responses against AMA1 VLPs Than TLA

**Figure 7:**
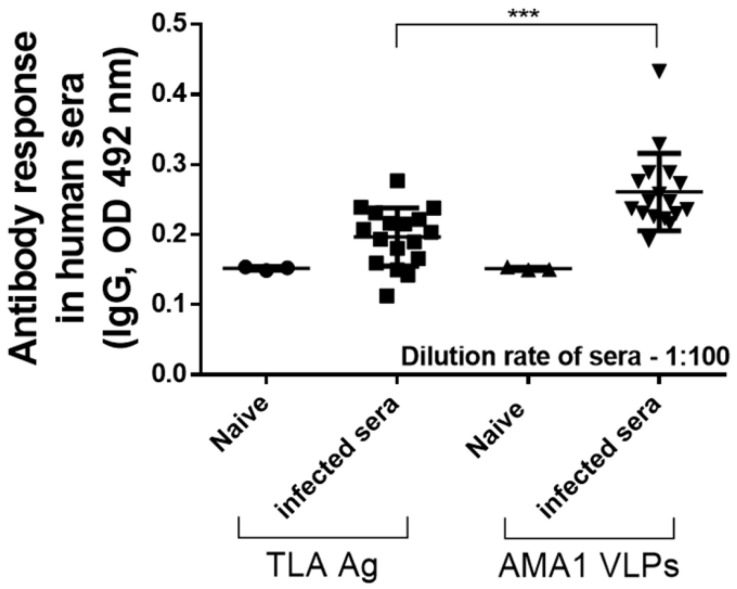
Reactivity of AMA1 VLPs antigen to *T. gondii*-infected human sera. To validate the serological findings observed from mice, human sera were acquired from toxoplasmosis patients. Toxoplasmosis-positive human serum IgG responses against TLA and AMA1 VLPs were compared to those of uninfected human sera. Data are presented as mean ± SD and are representative of the three individual experiments performed in triplicate (*** *p* < 0.001 vs. TLA).

**Figure 8:**
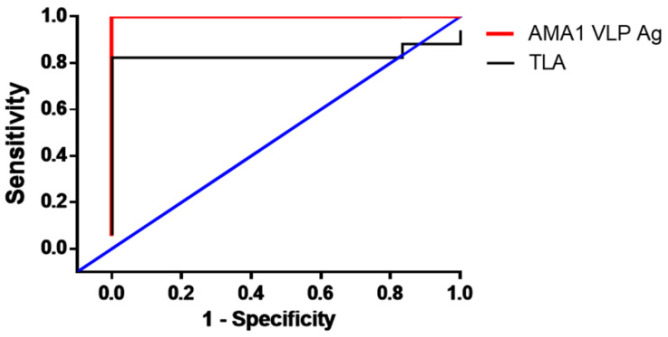
Comparison of receiver operating characteristics (ROC) curves. ROC analysis was used to compare the sensitivity and specificity of ELISA data acquired from AMA1 VLPs and TLA. The blue line shows a non-informative test curve.

To confirm whether the findings observed from murine sera were concordant with humans, toxoplasmosis-positive human sera (n = 17) were acquired and ELISA was performed. As expected, sera acquired from individuals with previous *T. gondii* exposure reacted against both TLA and AMA1 VLPs (Figure 7). Consistent with the murine sera ELISA results, the interaction of human sera was significantly stronger with AMA1 VLPs than with TLA (*** *p* < 0.001). Interestingly, there were several cases of false-negative results when ELISA was performed using TLA. Specifically, five patient samples had IgG OD values resembling that of naïve human sera results. Contrary to this, false-negative cases were not detected when sera were reacted with AMA1 VLPs. As indicated in Table 1 (Figure 8), AMA1 antigen-expressing AMA1 VLPs showed 90.91% sensitivity with 92.85% specificity, whereas lysate antigen (TLA) showed 74.72% sensitivity and 71.42% specificity.

Sensitivity and specificity of ELISA test for serodiagnosis of toxoplasmosis using TLA and AMA1 VLPs. The sensitivity and specificity of each antigen indicated in the table were calculated as described in the Material section and determined using human sera by ELISA.

## 4. Discussion

VLPs have been applied as serodiagnostic tools for several infectious diseases of viral origin [16,25,26]. Therefore, we hypothesized that this approach could also be applicable to parasitic diseases of global importance, such as toxoplasmosis. In the present study, we demonstrated that *T. gondii* AMA1 VLPs can sufficiently interact with toxoplasmosis-positive sera, thus revealing an alternative to the TLA or other recombinant antigens. Our findings revealed that potent IgM and IgG responses to both types I and II *T. gondii* clonal lineages could be elicited using AMA1 VLPs. Moreover, the responses were highly parasite-specific as cross-reaction with the rodent malaria sera was not observed. Human sera ELISA results were reflective of the data acquired from mice sera and confirmed the possibility of developing AMA1 VLPs as a serodiagnostic tool.

The major drawback of using TLA-based ELISA is the lack of specificity and sensitivity resulting from its preparation method. Given the inter-laboratory heterogeneity in the TLA preparation method, the antigen lysates are prone to contain various forms of contaminants originating from eukaryotic cells, culture media, or others. This in turn renders standardizing the TLA-based ELISA somewhat arduous. Resultantly, recombinant antigens have emerged to replace the conventional TLA as they are much easier to standardize [27]. To date, numerous antigens, either alone or mixed with other antigens, have been evaluated as toxoplasmosis serodiagnostic tools with varying sensitivity and specificity [27,28,29]. While many of these recombinant antigens have proven to be effective molecular markers for diagnosing toxoplasmosis, it is noteworthy to mention that these proteins were produced in *Escherichia coli*. Due to the fact that mammalian-like glycosylation and other PTMs are lacking in *E. coli*, this may have strong implications when it comes to an accurate diagnosis. For example, in the case of a *Strongyloides stercoralis* infection diagnosis, removing the carbohydrate moieties from antigens prior to ELISA-based serodiagnosis resulted in reduced seroreactivity, reduced optical density reading values, and increased the number of cross-reaction which contributed to erroneous results [30]. Unlike *E. coli*, VLPs produced in insect cells possess PTMs that are much closer to mammals, and therefore, carbohydrate-associated shortcomings would not affect serodiagnosis. This feature of the AMA1 VLPs may have contributed to the high specificity and sensitivity reported in our study. As with other organisms, a plethora of PTMs occurs in *T. gondii*. For example, both *N*- and *O*-linked glycosylations were observed in numerous *T. gondii* proteins including the rhoptries, micronemal proteins, surface antigens, and others [31]. Glycosylation is critical for proper protein folding and misfolded proteins or mutations in these aspects could alter the antigenicity of proteins. While investigating how closely insect cell PTMs resemble those of *T. gondii* is beyond the scope of this study, we speculate that these cellular processes are similar between the two. This notion is corroborated by the result of our previous *T. gondii* VLP vaccine studies [23,32,33], which demonstrated the protection elicited by insect cell-derived VLPs against *T. gondii* in mice.

Multiepitope-based recombinant antigen serodiagnosis is also emerging [34]. Here, Dai et al. reported 94.4% and 96.9% sensitivities for their multi-epitope serodiagnosis with 100% specificity. Using AMA1 antigen-expressing VLP, we confirmed 90.91% sensitivity with 92.85% specificity which are strikingly comparable to these values (Table 1). Much of our findings are consistent with previously reported results. *T. gondii* IgM titers were reported to span for months after infection [35]. In line with this finding, *T. gondii*-specific IgM responses against ME49 strains were detected even after 8 wpi. Similar results were detected for IgG as these were detected as early as 1 wpi and were induced to high levels. The specificity and sensitivity of the AMA1 VLPs closely resembled those of other recombinant antigens reported in the aforementioned studies [27,28,29]. Several studies have reported the presence of cross-interaction between *T. gondii* and other parasites, such as *Neospora caninum*, *Hamondia* spp., and *Eimeria* spp. [36,37]. AMA1 antigens of both *T. gondii* and *Plasmodium* spp. Share a highly conserved arrangement of 16 cysteine residues, which has a large influence on cross-reactivity [38,39]. Based on these previous reports, we initially anticipated that cross-reactivity between the two apicomplexan parasites *T. gondii* and *P. berghei* may occur to an extent. NCBI Protein BLAST analysis revealed 71% amino acid homology between AMA1 antigens of *T. gondii* (AF AF010264.1) and *P. berghei* (XM_034564734.1). Surprisingly, cross-reaction between the two was not detected at all and *P. berghei* immune sera failed to interact with *T. gondii* AMA1 VLPs. Meanwhile, all of the toxoplasmosis-positive sera reacted with the *T. gondii* AMA1 VLPs, thus signifying its high species-specificity. One of the noticeable differences in AMA1 of *T. gondii* and *Plasmodium* spp. is the PTM, especially palmitoylation. In the AMA1, a total of 18 cysteine residues are present: 16 conserved regions that likely form disulfide bridges, 1 in the signal peptide region, and the last cysteine residue located at position 504 near the cytoplasmic tail. However, the last cysteine residue where palmitoylation occurs in *T. gondii* is not conserved across multiple members of the *Plasmodium* spp., including *P. berghei* [40]. Investigating whether this difference accounts for the lack of cross-species interaction is beyond the scope of this study, but given that cysteine palmitoylation has a profound effect on immune-related aspects [41,42], it would be an interesting area to study in the future. Interestingly, human sample antibody responses were substantially lower than those elicited by murine sera. One factor accounting for this difference is the intensity of infection. Mice were infected with 450 cysts of *T. gondii* ME49 cysts, while human samples were acquired after natural infection with an unknown infection dose. Due to the fact that mice were infected with a higher dose of *T. gondii* than they would acquire upon natural exposure to the parasite, the antibody’s induction may have occurred to a greater extent. Consistent with this notion, a positive correlation between *T. gondii* ME49 infection dose and parasite-specific antibody induction in mice was reported in our previous study [43]. The duration of infection in humans also could have contributed to this difference. Although accurately discerning the time at which the patients were infected is difficult, it is presumed that the sera were acquired from chronic toxoplasmosis patients. If this is the case, the *T. gondii* parasite-specific antibodies circulating in hosts most probably waned over time, thereby resulting in low antibody responses.

Recombinant AMA1 has been evaluated as a serodiagnostic biomarker for toxoplasmosis. Recently, in a study by Ferra et al., the authors reported that the full-length AMA1 antigen demonstrated high specificity and sensitivity while serving as a better diagnostic indicator of toxoplasmosis than the N- or C-terminal AMA1 fragments [17]. Apart from the *T. gondii* strain used for infection, the results of Ferra et al. are similar to the findings observed in our study, thus further validating the diagnostic potential of AMA1 VLPs. AMA1 VLPs were highly *T. gondii*-specific and were capable of detecting multiple strains of *T. gondii* clonal lineages. Infections with RH and ME49, belonging to *T. gondii* type I and II clonal lineages respectively, were successfully detected using the AMA1 VLPs. However, clonal lineage type III and other exotic strains were not tested and this remains to be evaluated in the future. Additionally, given the small human toxoplasmosis serum samples, further studies need to be conducted with a greater sample size to increase statistical power and identify potential outliers.

One major limitation of ELISA and antibody-based serology testing is the fact that the exact time of infection cannot be accurately discerned. As with other serology-based assays, AMA1 VLPs reported in our study could not address whether the infection is at an acute or chronic stage, as strong IgM and IgG responses were elicited at both infection time points. However, there are several advantages to using AMA1 VLPs over TLA. First, AMA1 VLPs were capable of detecting *T. gondii* infection incurred by as low as ten cysts (Figure 1A). Although the cyst infection dose required to cause human toxoplasmosis remains unknown to this day [44], infection with ten cysts was enough to induce a significant increase in IgG antibody responses at 1 wpi. It is noteworthy to mention that significant levels of *T. gondii*-specific IgG response were observed as early as 1 wpi. This has important implications as it was reported from numerous human toxoplasmosis outbreaks that the parasite incubation period ranged from seven to thirty days [44]. Based on these results, developing AMA1 VLPs as a toxoplasmosis serodiagnostic tool could enable early and accurate diagnosis of toxoplasmosis which leads to proper patient treatment for their well-being. Overall, the *T. gondii* AMA1 VLPs antigen possesses higher specificity and sensitivity than TLA, and can also detect *T. gondii* infections at low doses. Further development and standardization of this antigen could enable rapid and accurate diagnosis of toxoplasmosis.

## 5. Conclusions

Virus-like particles displaying AMA1 of *T. gondii* can detect infections of both *T. gondii* ME49 and RH, even at an early stage of infection caused by very low infection doses in mice. High sensitivity and specificity were found using *T. gondii*-infected human sera, indicating AMA1 VLPs could be used for serological diagnosis of human toxoplasmosis.

## Figures and Tables

**Table 1:** Sensitivity and specificity of ELISA.

Antigen	Sensitivity	Specificity
*T. gondii* lysate antigen (TLA)	74.72%	71.42%
*T. gondii* AMA1 VLPs antigen	90.91%	92.85%

## Data Availability

The original contributions presented in this study are included in the article. Further inquiries can be directed to the corresponding author.
